# Assessing Stroke Caregivers′ Preparedness and Associated Factors for Patients′ Transition From Hospital to Home

**DOI:** 10.1155/srat/9657229

**Published:** 2026-08-26

**Authors:** Lisa A. Babkair, Mawaddah Abujabal, Maha Ali Basha, Manar Saeed, Rwan Mohammad Alyafee, Nora Jaber Alsolami, Salha Salem Algarni

**Affiliations:** ^1^ Critical Care Nursing Department, Faculty of Nursing, King Abdulaziz University, Jeddah, Saudi Arabia, kau.edu.sa; ^2^ Emergency Nursing Department, Faculty of Nursing, King Abdulaziz University, Jeddah, Saudi Arabia, kau.edu.sa; ^3^ Nursing Quality Coordinator of Surgical Department, King Fahad General Hospital, Jeddah, Saudi Arabia; ^4^ Nursing Quality Coordinator, King Abdullah Medical Complex, Jeddah, Saudi Arabia

**Keywords:** caregivers, preparedness, stroke, stroke survivors, transition home

## Abstract

**Introduction:**

Stroke not only affects stroke survivors but also places considerable demands on family caregivers. Inadequate preparedness to provide care may contribute to caregiver burden and challenges during the transition from hospital to home

**Objective:**

This study is aimed at assessing caregiver preparedness and identify factors associated with preparedness among family caregivers of stroke survivors during the transition from hospital to home.

**Methods:**

A descriptive cross‐sectional study was conducted among 132 family caregivers of stroke survivors in two hospitals in Jeddah, Saudi Arabia. Data were collected using a sociodemographic questionnaire, the Preparedness for Caregiving Scale (PCS), and the 12‐item Zarit Burden Interview (ZBI‐12). Normality was assessed using the Kolmogorov–Smirnov and Shapiro–Wilk tests. Nonparametric analyses and multiple linear regression were performed.

**Results:**

More than half of the caregivers (57.6%) reported low preparedness, whereas 51.5% experienced high caregiver burden. The median PCS score was 16.0 (IQR = 12), and the median caregiver burden score was 21.0 (IQR = 15). Preparedness was positively associated with stroke onset duration (*ρ* = 0.832, *p* < 0.001) and negatively associated with caregiver burden (*ρ* = −0.812, *p* < 0.001). Female caregivers reported higher preparedness scores than male caregivers (*p* < 0.001). Multiple linear regression demonstrated that stroke onset duration, caregiver burden, and caregiver gender were independent predictors of preparedness.

**Conclusion:**

Family caregivers of stroke survivors frequently experience low preparedness and high caregiver burden during the transition from hospital to home. Early assessment of caregiver preparedness and burden may facilitate targeted interventions to support caregivers during the post‐stroke transition period.

## 1. Introduction

Stroke remains the second leading cause of death and the third leading cause of disability globally [1]. According to the World Stroke Organization, 12.2 million new strokes occur annually, and over 101 million people are currently living with the effects of stroke—a number that has doubled over the past 30 years. Stroke is a devastating disease that affects both survivors and their caregivers. Caregivers who are unprepared to provide direct care to stroke survivors often experience stress, depression, fatigue, and a sense of burden due to a lack of knowledge and experience in caregiving [2, 3]. This lack of preparedness can result in difficulties in managing stroke survivors, leading to unmet medical needs and an increased risk of safety concerns [4]. Consequently, patients are more likely to face complications or require additional medical interventions, raising the likelihood of hospital readmission. As [5] highlight, these challenges not only elevate the risk of readmissions but also drive up healthcare costs, emphasizing the importance of addressing caregiver preparedness to prevent avoidable hospitalizations. Most stroke survivors experience varying degrees of physical, cognitive, and functional impairment, often requiring ongoing assistance from family caregivers following hospital discharge [6, 7]. In Saudi Arabia, the incidence of stroke is rising. Recent studies indicate that 30–40 new stroke cases occur annually for every 1000 people, with a prevalence rate of 186 per 100,000 [8]. Over the past decade, the population has grown by 12.8%, with the estimated rate of first strokes ranging from 57% to 67%, depending on the data used [9]. Despite the increasing number of stroke cases, access to rehabilitation and community‐based support services remains variable across regions in Saudi Arabia. Following hospital discharge, family caregivers often play a central role in supporting stroke survivors with daily care needs, medication management, mobility assistance, and follow‐up care. Therefore, adequate caregiver preparation is essential to facilitate a safe and effective transition from hospital to home. In Saudi Arabia, family members are traditionally expected to assume a central role in caring for relatives with chronic illnesses and disabilities. These caregiving responsibilities are influenced by cultural values emphasizing family obligation and collective support. Consequently, caregivers may face unique challenges and support needs during the transition from hospital to home. Understanding caregiver preparedness within this sociocultural context is essential for developing effective discharge planning strategies and culturally appropriate caregiver support programs.

Several studies have identified factors that may influence caregiver preparedness, such as educational level, profession, caregiving experience, and the caregiver′s gender and age [4, 10]. Based on previous studies, the preparedness of family caregivers to care for stroke survivors transitioning from hospital to home requires further attention [4]. Caregiver preparedness is defined as the perceived preparedness of caregivers to meet the physical and emotional needs of the patient [11]. Furthermore, [12] and colleagues conducted a concept analysis to clarify the meaning of preparedness for informal caregivers of older adults. They defined the concept of preparedness of informal caregivers as caregiver′s self‐confidence about their current competence related to the knowledge, skills and abilities to perform daily tasks, and to handle emotions over time .

Previous studies have shown that caregivers of stroke survivors often report substantial burden and inadequate preparedness to meet survivors′ physical, cognitive, and emotional needs [13, 14]. Caregiver preparedness is associated with both caregiver well‐being and patient outcomes, whereas inadequate preparation may contribute to caregiver stress, increased burden, and poorer transition experiences following hospital discharge [4, 10].

Stroke places a significant burden on caregivers, particularly in the first few weeks following discharge. Caregivers of stroke patients report high levels of burden due to the increased demands of daily caregiving tasks [15]. This burden has negative consequences for both caregivers and patients, including an increased risk of patient morbidity and elevated levels of perceived stress [15]. Some factors affecting caregivers preparedness include the fear of stroke disrupting a normal lifestyle, the transition to an unfamiliar environment, and the process of adapting to a survivor′s disabilities [16, 17]. Additionally, caregivers with prior caregiving experience demonstrate greater psychological preparedness to assist with patient rehabilitation and meet the survivor′s needs [4].

To our knowledge, few studies have examined stroke caregiver preparedness and associated factors in Saudi Arabia. Although caregiver preparedness has been investigated in several Western countries, caregiver experiences may be influenced by cultural norms, family structures, and healthcare system characteristics that differ across regions. Consequently, findings from Western settings may not be fully transferable to the Saudi context. Examining caregiver preparedness in Saudi Arabia is important for generating culturally relevant evidence to guide caregiver education, discharge planning, and supportive interventions. The purpose of this study was to assess caregiver preparedness and identify factors associated with preparedness among family caregivers of stroke survivors during the transition from hospital to home.

## 2. Materials and Methods

A quantitative, descriptive, cross‐sectional design was used to conduct this study, with data collected at a single point in time. This study was conducted at two hospitals in Jeddah, Saudi Arabia. These hospitals were selected because both are eligible to admit stroke patients and provide stroke care. A convenience sample of 132 caregivers of stroke survivors was selected from the aforementioned settings to participate in this study. Family caregivers were eligible to participate if they were providing unpaid care to a stroke survivor who had experienced a stroke within the previous 4 months. Eligible caregivers were required to be at least 18 years of age, be a first‐degree relative of the stroke survivor (e.g., spouse, parent, son, or daughter), reside in the same household as the survivor and be able to communicate in Arabic. To ensure independence of observations, only one primary caregiver was recruited for each stroke survivor. When more than one family member was involved in caregiving, the caregiver identified as having the greatest caregiving responsibility was invited to participate. Both Saudi and non‐Saudi caregivers were eligible to participate. Non‐Saudi caregivers were included because they resided in Saudi Arabia, received services within the same healthcare system, and provided care within a similar sociocultural environment. All participants were Arabic‐speaking family caregivers caring for stroke survivors living in Saudi Arabia. Family caregivers who did not provide consent were excluded from the study. Additionally, caregivers with physical or cognitive impairments that hindered their participation, as well as professional or paid caregivers, were also excluded.

**Figure 1:**
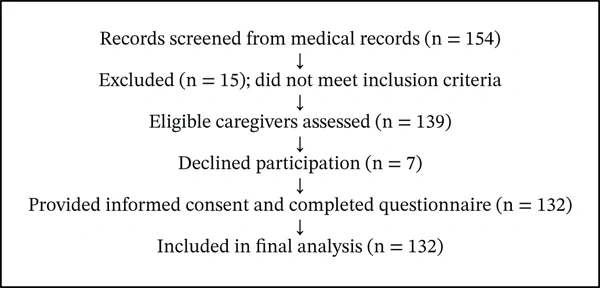
Participant recruitment flow diagram.

Potential participants were identified through medical records of selected hospitals. A total of 154 stroke survivor records were screened to identify patients who had experienced a stroke within the previous 4 months. Following screening, 139 family caregivers were assessed for eligibility according to the study inclusion and exclusion criteria. Of these, 15 caregivers were excluded because they did not meet the eligibility criteria. Eligible caregivers were contacted by telephone, provided with information about the study, and invited to participate. Seven caregivers declined participation. Caregivers who met the eligibility criteria and agreed to participate provided electronic informed consent before completing the study questionnaires through SurveyMonkey during a researcher‐assisted telephone interview. A total of 132 caregivers completed the survey and were included in the final analysis (Figure 1). No missing data were observed because all questionnaire items were mandatory within the SurveyMonkey platform and participants were guided through survey completion by the researchers.

The questionnaire used in this study consists of three parts: general information, Preparedness for Caregiving Scale (PCS), and the 12‐item Zarit Burden Interview (ZBI‐12). The general information questionnaire is designed to gather essential demographic data from participants in the study. It includes variables potentially influencing caregiver preparedness, such as age, gender, nationality, marital status, number of household members, literacy level, medical history, time since hospital discharge, and monthly income. Stroke onset duration was also collected and defined as the time elapsed since the stroke event, measured in months. To focus on the early transition period from hospital to home, only caregivers of stroke survivors who had experienced a stroke within the previous 4 months were eligible to participate. Accordingly, stroke onset duration ranged from 1 to 4 months, and caregivers of stroke survivors whose stroke onset exceeded 4 months were excluded from the study.

The PCS is a self‐rated caregiver tool comprising eight items that ask caregivers how prepared they believe they are for different aspects of caregiving. “Preparedness” refers to the perceived preparedness to provide emotional and physical support, arrange in‐home assistance, and manage caregiving‐related stress. Responses are rated on a five‐point scale from 0 (*Not at all prepared*) to 4 (*Very well prepared*). The mean of all items was calculated, with scores ranging from 0 to 4 and the total score ranges from 0 to 32 with higher scores indicating a higher level of preparedness for caregiving [11]. The PCS is a valid and reliable tool for assessing caregiving preparedness in caregivers of stroke survivors. It is a useful instrument for healthcare providers to identify caregivers who may require specific interventions to improve their preparedness for caregiving tasks [11]. The reliability of the scale demonstrated excellent internal consistency with Cronbach′s alpha of 0.94 and the item‐reliability index and item‐total association were above 0.30. Furthermore, the test‐retest reliability was found to be 0.92, demonstrating the stability of the scale over time when administered to the same participants.

The 12‐item ZBI‐12 was used to assess caregiver burden. The ZBI‐12 is a shortened version of the original ZBI‐12 developed by Zarit et al. (1980) and later adapted by Bédard et al. (2001) [18, 19]. The instrument evaluates the perceived impact of caregiving on caregivers′ emotional, physical, social, and psychological well‐being. Each item is rated on a 5‐point Likert scale ranging from 0 (*Never*) to 4 (*Nearly Always*), yielding a total score ranging from 0 to 48, with higher scores indicating greater caregiver burden. Scores of 0–10 indicate no or mild burden, scores of 11–20 indicate mild to moderate burden, and scores greater than 20 indicate high caregiver burden. The ZBI‐12 has demonstrated good reliability and validity in previous studies. Bédard et al. (2001) reported good internal consistency for the English version, with a Cronbach′s alpha coefficient of 0.88 [19].

The Arabic abridged version of the ZBI (ZBI‐A) has demonstrated satisfactory psychometric properties among Arabic‐speaking caregivers. Bachner (2013) reported acceptable internal consistency, with a Cronbach′s alpha coefficient of 0.90, supporting the reliability of the instrument. The study also demonstrated satisfactory construct validity, indicating that the Arabic version is a reliable and valid measure of caregiver burden. The Arabic version of the ZBI‐A was used in the present study [20].

### 2.1. Statistical Analysis

A priori power analysis was conducted using G ^∗^Power to determine the sample size required to address the research questions. The analysis indicated that a minimum sample size of 80 participants was required to achieve 80% power for detecting a medium effect size at a significance level of *α* = 0.05. Recruitment continued throughout the study period, and all eligible caregivers who met the inclusion criteria and agreed to participate were enrolled. Consequently, a total of 132 caregivers were included in the final analysis. The larger sample size increased statistical precision and enhanced the robustness of the study findings.

Prior to inferential analyses, the normality of the PCS and caregiver burden scores was assessed using the Kolmogorov–Smirnov and Shapiro–Wilk tests. Both PCS scores (Shapiro–Wilk = 0.962, *p* = 0.001) and caregiver burden scores (Shapiro–Wilk = 0.976, *p* = 0.020) significantly deviated from a normal distribution. Therefore, nonparametric statistical tests were used for group comparisons. Mann–Whitney *U* tests were applied to compare preparedness scores between two groups, whereas Kruskal–Wallis tests were used for variables with more than two categories. Spearman correlation coefficients were used to examine associations between continuous variables and preparedness scores. Variables significantly associated with preparedness in the bivariate analyses were subsequently entered into a multiple linear regression model to identify independent predictors of caregiver preparedness.

### 2.2. Ethical Considerations

Ethical approval was granted by the Faculty of Nursing as well as by the Ministry of Health with code number: NREC Serial No: Ref No 2B. 67. All participants were informed of the study purpose and invited to participate at their convenience. Informed consents were obtained from all participants after a detailed explanation of the research. Although researchers assisted participants during questionnaire completion and were aware of participant identities for recruitment purposes, no identifying information was linked to survey responses during data analysis. All questionnaire responses were deidentified prior to analysis to maintain participant confidentiality. All questions were required to be answered for successful submission through the SurveyMonkey platform. They were also informed that they were free to refuse participation if they felt uncomfortable. All data were kept confidential and only accessible to the researchers. This study complies with the Strengthening the Reporting of Observational Studies in Epidemiology (STROBE) guidelines for cross‐sectional studies, as specified in the EQUATOR checklist [21].

## 3. Results

A sample of 132 caregivers of stroke patients was enrolled. The sample included caregivers of patients who had been diagnosed with stroke within the last 4 months. Table 1 presents the sociodemographic characteristics of the sample. The mean age of the caregivers was 35.34 years (SD = 12.49). The average number of caregivers living in the household with patients was 4.81 people (IQR = 2). Among stroke survivors, the mean time since stroke onset was 2.47 months (SD = 0.90), with all participants falling within the study eligibility range of 1–4 months. The majority of the caregivers were Saudi (*n* = 109, 82.6%), female (*n* = 70, 53%), and married (*n* = 77, 58.3%). Most of the sample had a university‐level education (*n* = 89, 67.40%), and more than half were employed (*n* = 69, 52.30%). Regarding socioeconomic status, over one‐third of the sample (*n* = 52, 39.40%) had a monthly income of more than 10,000 Saudi Riyals; approximately 2600 USD/month. Regarding medical conditions of caregiver sample, the most common comorbidities among caregivers were hypertension (*n* = 27, 20.50%), hyperlipidemia (*n* = 24, 18.20%), diabetes (*n* = 22, 16.70%), and smoking (*n* = 23, 17.40%).

**Table 1:** Characteristics of family caregivers and stroke survivors (*n* = 132).

	Minimum	Maximum	Mean/median	Std. deviation/IQR

Age, years (mean/Std. deviation)	20	74	35.34	12.49
Number of people live in house (median/IQR)	2	9	5	2
Stroke onset duration, months (mean/Std. deviation)	1	4	2.47	0.91

**Characteristics**			**Caregivers (** **n** = 132 **)**	
			**N**	**(%)**

Sex	Male		62	47
	Female		70	53
Nationality	Saudi		109	83
	Non‐Saudi		23	17
Marital status	Married		77	58
	Single		45	34
	Divorced		4	3.0
	Widow		6	5.0
Education	Elementary school		2	2.0
	Middle school		3	2.0
	High school		28	21
	University		90	68
	Higher degree		9	7.0
Employment	Employed		69	52
	Unemployed		23	18
	Retired		15	11
	Housewife		25	19
Monthly salary (Saudi Riyal)	< 2000		15	11
	2000–5000		25	19
	5000–10,000		30	23
	> 10,000		52	39
	Do not know		10	8.0
Medical conditions	Diabetes mellitus		17	13
	Hypertension		31	23
	Heart disease		2	2.0
	Kidney disease		2	2.0
	Hyperlipidemia		24	18
	Obesity		4	3.0
	Smoking		27	20
	Non		39	30
Relationship with a stroke survivor	Father		23	17
	Mother		29	22
	Siblings		17	13
	Husband		11	8.0
	Wife		38	29
	Other		14	11

Assessment of data distribution demonstrated that both preparedness and caregiver burden scores were not normally distributed. Therefore, median and interquartile range (IQR) are presented as the primary descriptive statistics, whereas means and standard deviations are also reported to facilitate comparison with previous studies. The median PCS score was 16.00 (IQR = 12), indicating a generally low level of preparedness among caregivers. The median caregiver burden score was 21.00 (IQR = 15), reflecting a high level of caregiver burden. Detailed descriptive statistics for the PCS and ZBI‐12 scores are presented in Table 2.

**Table 2:** Caregivers preparedness and burden scores (*n* = 132).

	*N*	Median (IQR)	Mean (Std. deviation)
PCS total	132	16.00 (12)	16.96 (7.80)
Burden total score	132	21.00 (15)	19.68 (10.3)

*Note:* PCS total scores < 19 represent low preparedness, scores 19–23 represent average preparedness, and scores ≥ 24 represent high preparedness. Burden score 0–10 represents no or mild burden, 10–20 represents mild to moderate, and > 20 represents high burden.

Abbreviation: IQR, interquartile range.

Table 3 shows the PCS subcategories classified into low preparedness, average preparedness, and high preparedness. About 57% of the sample showed a low preparedness level for caring for stroke survivors after transition from hospital to home.

**Table 3:** The Preparedness Caregivers Scale (PCS) subcategories.

Subcategories	Caregivers (*n* = 132)	
	*n*	%
Low preparedness	76	57
Average preparedness	22	17
High preparedness.	34	26

*Note:* Scores < 19 represent low preparedness, scores 19–23 represent average preparedness, and scores ≥ 24 represent high preparedness.

Table 4 shows the caregiver burden ZBI‐12 subcategories classified into low or no burden, mild to moderate burden, and high burden. About 52% of the sample showed a high burden level for caring for stroke survivors after transition from hospital to home.

**Table 4:** Caregiver burden ZBI‐12 subcategories.

Subcategories	Caregivers (*n* = 132)	
	*n*	%
No burden	28	21
Mild to moderate burden	36	27
High burden	68	52

*Note:* Scores 0–10 represent no or low burden, scores 10–20 represent mild to moderate burden, and scores ≥ 20 represent high burden.

Spearman correlation analysis demonstrated a significant positive association between caregiver age and preparedness scores (*ρ* = 0.617, *p* < 0.001). Likewise, stroke onset duration showed a strong positive correlation with caregiver preparedness (*ρ* = 0.832, *p* < 0.001). A strong negative correlation was observed between caregiver burden and preparedness scores (*ρ* = −0.812, *p* < 0.001), indicating that caregivers with higher preparedness reported lower levels of caregiver burden. However, no significant association was found between household size and preparedness scores (*ρ* = 0.101, *p* = 0.250). A Mann–Whitney *U* test revealed a significant difference in preparedness scores according to caregiver gender (*Z* = −4.532, *p* < 0.001), with female caregivers demonstrating higher preparedness scores than male caregivers. No significant difference was observed according to caregiver nationality (*Z* = −0.649, *p* = 0.517). Kruskal–Wallis tests indicated that caregiver preparedness did not significantly differ according to marital status (*H* = 1.294, *p* = 0.731), educational level (*H* = 2.784, *p* = 0.595), employment status (*H* = 6.625, *p* = 0.085), or household monthly income (*H* = 5.394, *p* = 0.145). Detailed results are presented in Table 5.

**Table 5:** Associations between caregiver characteristics and preparedness for caregiving scores.

Variable	Statistical test	Statistic	*p*
Age	Spearman correlation	*ρ* = 0.617	< 0.001 ^∗^
Household size	Spearman correlation	*ρ* = 0.101	0.250
Stroke onset duration	Spearman correlation	*ρ* = 0.832	< 0.001 ^∗^
Caregiver burden	Spearman correlation	*ρ* = −0.812	< 0.001 ^∗^
Gender	Mann–Whitney U	*Z* = −4.532	< 0.001 ^∗^
Nationality	Mann–Whitney U	*Z* = −0.649	0.517
Marital status	Kruskal–Wallis	*H* = 1.294	0.731
Educational level	Kruskal–Wallis	*H* = 2.784	0.595
Employment status	Kruskal–Wallis	*H* = 6.625	0.085
Household monthly income	Kruskal–Wallis	*H* = 5.394	0.145

*Note: ρ* = Spearman′s rank correlation coefficient; Z = Mann–Whitney *U* test statistic; H = Kruskal–Wallis test statistic.

^∗^ *p* < 0.5 indicates statistical significance.

A multiple linear regression analysis was conducted to identify factors independently associated with caregiver preparedness as presented in Table 6. The overall regression model was statistically significant (*F* = 126.296, *p* < 0.001) and explained 80.0% of the variance in preparedness scores (*R* ^2^ = 0.800; adjusted *R* ^2^ = 0.794). Prior to model interpretation, multicollinearity was assessed using variance inflation factors (VIFs) and tolerance values. The results indicated no evidence of problematic multicollinearity among the predictor variables (all VIFs < 5).

**Table 6:** Multiple linear regression analysis of factors associated with caregiver preparedness.

Predictor	*B*	SE	*β*	*t*	*p*
Age	0.023	0.031	0.037	0.757	0.450
Gender	2.272	0.650	0.146	3.494	0.001 ^∗^
Stroke onset duration	3.810	0.568	0.442	6.709	< 0.001 ^∗^
Burden total score	−0.336	0.043	−0.443	−7.776	< 0.001 ^∗^

*Note: B* = unstandardized regression coefficient; SE = standard error; *β* = standardized regression coefficient. Model statistics: *R* ^2^ = 0.800; adjusted *R* ^2^ = 0.794; *F* (4,126) = 126.296; *p* < 0.001.

^∗^ *p* < 0.5 indicates statistical significance.

Stroke onset duration (*β* = 0.442, *p* < 0.001), caregiver burden (*β* = −0.443, *p* < 0.001), and caregiver gender (*β* = 0.146, *p* = 0.001) remained independently associated with preparedness scores. Longer stroke duration and female gender were associated with higher preparedness, whereas greater caregiver burden was associated with lower preparedness. Caregiver age was not independently associated with preparedness after adjustment for the other variables (*β* = 0.037, *p* = 0.450).

## 4. Discussion

The present study investigated caregiver preparedness and associated factors among family caregivers of stroke survivors during the transition from hospital to home. The findings demonstrated that most caregivers reported low levels of preparedness and high levels of caregiver burden during the early post‐stroke period. Bivariate analyses showed that caregiver age, stroke onset duration, caregiver burden, and gender were significantly associated with preparedness. However, multiple linear regression analysis revealed that stroke onset duration, caregiver burden, and caregiver gender remained independent predictors of preparedness, whereas caregiver age was no longer significant after adjustment. These findings suggest that caregiving experience, caregiver burden, and gender‐related caregiving roles play important roles in shaping caregiver preparedness during the transition from hospital to home.

The present study found that most caregivers reported low levels of preparedness during the transition from hospital to home, with more than half of the caregivers classified as having low preparedness. These findings are consistent with previous studies that have reported inadequate preparedness among caregivers of stroke survivors. Liu et al. (2020) reported a mean PCS score of 14.42, indicating poor preparedness among family caregivers, whereas other studies reported similarly low preparedness scores ranging from 18.55 to 20.07 [4, 10].

The consistently low preparedness observed across studies may be explained by the sudden onset of stroke and the rapid transition from hospital to home. Many stroke survivors experience substantial physical, cognitive, and functional impairments that require ongoing assistance with daily activities, medication management, and rehabilitation. Family caregivers often assume these responsibilities with limited prior caregiving experience and insufficient preparation. Previous studies have shown that adequate caregiver preparedness contributes to improved caregiver well‐being and better patient outcomes, including enhanced functional recovery and quality of life among stroke survivors [3].

In the present study, caregiver age was positively associated with preparedness in the bivariate analysis; however, age did not remain an independent predictor in the multivariable model. Previous studies have similarly reported associations between caregiver age and preparedness. Gutierrez‐Baena and Romero‐Grimaldi (2022) reported a significant association between age and caregiver preparedness, whereas Septianingrum et al. (2024) found that younger caregivers demonstrated lower preparedness levels [10, 22].

The loss of statistical significance after adjustment suggests that the influence of age may be explained by other factors, such as caregiving experience and caregiver burden. Younger caregivers may experience competing educational, occupational, and family responsibilities, which can influence their ability to adapt to caregiving demands [23]. However, the present findings indicate that age alone may not independently determine caregiver preparedness when other caregiving‐related factors are considered.

Caregiver gender was independently associated with preparedness, with female caregivers reporting higher preparedness levels than male caregivers. This finding remained significant in the multivariable regression analysis, suggesting that gender plays an important role in caregiver preparedness. Previous studies have similarly reported an association between caregiver gender and preparedness. Gutierrez‐Baena and Romero‐Grimaldi (2022) found a significant relationship between gender and preparedness, whereas Septianingrum et al. (2024) identified gender as one of the strongest predictors of caregiver preparedness [10, 22].

The higher preparedness observed among female caregivers may reflect traditional caregiving roles and sociocultural expectations. In Saudi Arabia and many other societies, women often assume primary responsibility for caring for ill family members and may therefore have greater involvement in caregiving activities and decision‐making. Previous studies have suggested that women may experience greater emotional involvement and caregiving engagement, which could contribute to increased confidence and preparedness for caregiving responsibilities [24]. The persistence of gender as an independent predictor in the present study highlights the importance of considering gender‐related caregiving roles when developing caregiver education and support interventions.

Stroke onset duration was positively associated with caregiver preparedness and remained an independent predictor in the multivariable analysis. Caregivers of stroke survivors with a more recent stroke onset reported lower preparedness levels, whereas longer stroke duration was associated with greater preparedness. These findings are consistent with those reported by Gutierrez‐Baena and Romero‐Grimaldi (2022), who found that longer caregiving duration was associated with higher preparedness levels [10].

The relationship between stroke duration and preparedness may reflect the gradual adaptation of caregivers to their caregiving responsibilities. During the early period following hospital discharge, caregivers often face unfamiliar caregiving tasks, emotional stress, and uncertainty regarding patient recovery. As caregiving experience increases over time, caregivers may acquire knowledge, skills, and confidence that improve their preparedness to manage the physical, emotional, and practical demands of caregiving [4]. The present findings emphasize the importance of providing caregiver education and support early in the transition from hospital to home, particularly during the first months following stroke.

An important finding of the present study was the strong inverse relationship between caregiver burden and preparedness. Higher levels of caregiver burden were associated with lower preparedness scores, and caregiver burden remained an independent predictor of preparedness after adjustment for other variables. More than half of the caregivers in this study experienced high levels of burden, highlighting the substantial challenges encountered during the transition from hospital to home.

These findings are consistent with recent evidence reported by Ding et al. (2025), who found that informal caregivers of stroke survivors experienced the lowest preparedness and the highest burden during the early transition period, with preparedness increasing and burden decreasing over time [25]. Similarly, Uhm et al. (2023) reported that higher caregiver preparedness was significantly associated with lower caregiver burden, lower depression symptoms, and better quality of life among caregivers of individuals with disabilities, the majority of whom were caring for stroke survivors. The strong negative association observed in the present study further supports the close relationship between preparedness and caregiver burden [26].

The relationship between preparedness and burden may be explained by the emotional, physical, and psychological demands associated with caregiving. Caregivers who feel inadequately prepared may experience greater stress, uncertainty, and difficulty managing caregiving responsibilities, particularly during the early postdischarge period. Previous studies have shown that caregivers often experience insufficient caregiving knowledge, inadequate discharge information, and uncertainty regarding the recovery process during the transition from hospital to home [4]. Conversely, caregivers with greater knowledge, confidence, and caregiving skills may be better equipped to cope with caregiving demands and adapt to their new roles.

These findings emphasize the importance of early caregiver assessment, education, and supportive interventions during discharge planning. Improving caregiver preparedness may not only enhance caregivers′ confidence and competence but may also reduce caregiver burden and facilitate a smoother transition from hospital to home.

In the present study, caregiver preparedness was not significantly associated with caregiver nationality, marital status, educational level, employment status, household income, or the number of individuals living in the household. Similarly, none of these variables emerged as independent predictors in the multivariable analysis. However, Septianingrum et al. (2024) reported significant associations between preparedness and marital status, socioeconomic status, and employment status. Previous studies have also reported inconsistent findings regarding the relationship between educational level and caregiver preparedness [22].

The absence of significant associations in the present study suggests that caregiving‐related factors, such as caregiver burden and caregiving experience, may have a greater influence on preparedness than sociodemographic characteristics alone. These findings highlight the importance of focusing on caregivers′ experiences and support needs rather than relying solely on demographic characteristics when assessing caregiver preparedness [4].

The findings of this study provide important implications for nursing practice and transitional care services in Saudi Arabia. Family caregivers frequently experienced low preparedness and high levels of caregiver burden during the early post‐stroke period, highlighting the need for systematic caregiver assessment during the transition from hospital to home. Healthcare professionals, particularly nurses, should assess both caregiver preparedness and caregiver burden before discharge to identify caregivers who may require additional support.

The identification of caregiver burden, stroke onset duration, and caregiver gender as independent predictors of preparedness suggests that caregiver support interventions should be individualized according to caregivers′ specific needs and circumstances. Caregivers experiencing high burden, caregivers of recently discharged stroke survivors, and caregivers with lower preparedness levels may particularly benefit from targeted educational and supportive interventions.

These findings emphasize the importance of integrating caregiver assessment into discharge planning and transitional care programs. Providing individualized education, practical caregiving training, and emotional support may improve caregivers′ confidence, reduce caregiver burden, and facilitate a safer and more successful transition from hospital to home. Future research should evaluate interventions designed to improve caregiver preparedness and reduce caregiver burden among family caregivers of stroke survivors.

## 5. Limitations

This study has several limitations. First, family caregivers were recruited using a convenience sampling method, which may limit the generalizability of the findings. Second, the cross‐sectional design and the collection of data at a single time point preclude causal inferences between caregiver preparedness, caregiver burden, and associated factors. Third, the study was conducted in two hospitals within one region of Saudi Arabia, which may limit the applicability of the findings to other healthcare settings and geographical areas. Finally, caregiver preparedness and caregiver burden were assessed using self‐reported measures, which may be subject to response bias. In addition, the use of researcher‐assisted telephone interviews may have introduced social desirability bias, whereby participants may have provided responses that they perceived to be more socially acceptable. Despite these limitations, the study provides important insights into caregiver preparedness and caregiver burden among family caregivers of stroke survivors during the transition from hospital to home.

## 6. Conclusions

This study demonstrated that family caregivers of stroke survivors frequently experience low levels of preparedness and high levels of caregiver burden during the transition from hospital to home. Longer stroke duration, female gender, and lower caregiver burden were independently associated with higher preparedness levels. These findings suggest that caregiver burden and caregiving experience play important roles in shaping caregiver preparedness. Healthcare professionals should assess caregiver preparedness and caregiver burden early during the post‐stroke transition period and provide individualized educational and supportive interventions to address caregivers′ needs. Future research should evaluate interventions aimed at improving caregiver preparedness and reducing caregiver burden among family caregivers of stroke survivors.

## Funding

No funding was received for this manuscript.

## Conflicts of Interest

The authors declare no conflicts of interest.

## Supporting information


**Supporting Information** Additional supporting information can be found online in the Supporting Information section. 

## Data Availability

The data that support the findings of this study are available from the corresponding author upon reasonable request.
